# Dynamic changes of CX3CL1/CX3CR1 axis during microglial activation and motor neuron loss in the spinal cord of ALS mouse model

**DOI:** 10.1186/s40035-018-0138-4

**Published:** 2018-12-21

**Authors:** Jingjing Zhang, Yufei Liu, Xinyao Liu, Song Li, Cheng Cheng, Sheng Chen, Weidong Le

**Affiliations:** 1 Center for Clinical Research on Neurological Diseases, the First Affiliated Hospital, Dalian Medical University, Dalian, 116021 People’s Republic of China; 2 Liaoning Provincial Key Laboratory for Research on the Pathogenic Mechanisms of Neurological Diseases, the First Affiliated Hospital, Dalian Medical University, Dalian, 116021 People’s Republic of China; 3 Chifeng Municipal Hospital, Chifeng, 024000 People’s Republic of China; 4 Department of Neurology, Ruijin Hospital, Shanghai Jiao Tong University School of Medicine, Shanghai, 200025 China

**Keywords:** ALS, CX3CL1/CX3CR1 axis, Microglial activation, SOD1^G93A^ mice

## Abstract

**Background:**

Neuron-microglia communication plays a crucial role in the motor neurons (MNs) death in amyotrophic lateral sclerosis (ALS). Neurons can express chemokine (C-X3-C motif) ligand 1 (CX3CL1), which mediates microglial activation via interacting with its sole receptor CX3CR1 in microglia. In the present study, we aimed to investigate the dynamic changes of CX3CL1/CX3CR1 axis during microglial activation and MNs loss in SOD1^G93A^ mouse model of ALS.

**Methods:**

qPCR, western blot and immunofluorescent staining were used to examine the mRNA and protein levels and localization of CX3CL1/CX3CR1 in the anterior horn region of spinal cord in both SOD1^G93A^ mice and their age-matched wild type (WT) littermates at 40, 60, 90 and 120 days of age. The M1/M2 microglial activation in the spinal cord tissues of SOD1^G93A^ mice and WT mice were evaluated by immunofluorescent staining of M1/M2 markers and further confirmed by qPCR analysis of M1/M2-related cytokines.

**Results:**

The immunofluorescent staining revealed that CX3CL1 was predominately expressed in MNs, while CX3CR1 was highly expressed in microglia in the anterior horn region of spinal cord. Compared with age-matched WT mice, CX3CL1 mRNA level was elevated at 40 days but decreased at 90 and 120 days in the anterior horn region of spinal cords in ALS mice. Consistently, CX3CR1 mRNA level was increased at 90 and 120 days. Western blot assay further confirmed the dynamic changes of CX3CL1/CX3CR1 axis in ALS mice. Additionally, the levels of M1/M2 markers of microglia and their related cytokines in the anterior horn region of spinal cord in ALS mice were increased at 90 and 120 days. Moreover, while M1-related cytokines in ALS mice were persistently increased at 120 days, the upregulated M2-related cytokines started to decline at 120 days, suggesting an altered microglial activation.

**Conclusions:**

Our data revealed the dynamic changes of CX3CL1/CX3CR1 axis and an imbalanced M1/M2 microglial activation during ALS pathological progression. These findings may help identify potential molecular targets for ALS therapy.

**Electronic supplementary material:**

The online version of this article (10.1186/s40035-018-0138-4) contains supplementary material, which is available to authorized users.

## Introduction

Amyotrophic lateral sclerosis (ALS) is a chronic neurodegenerative disease characterized by the loss of both lower and upper motor neurons (MNs). Most ALS patients present progressive muscle weakness and atrophy, and eventually died within 5 years of the disease onset. At present, no therapy could delay the disease progression except riluzole and edaravone, which can extend some ALS patients’ lifespan for only a few months. The etiology and pathogenesis of ALS are believed to be multifactorial, and the exact mechanisms have not been fully understood. Most ALS cases are sporadic, while around 10% of patients are familial. Identification of pathogenic genes for familial ALS has facilitated the research for key pathogenic mechanisms of this disease [1]. Cu/Zn superoxide dismutase 1 (SOD1) was the first gene identified to be associated with ALS, accounting for about 10–20% of familial ALS. SOD1^G93A^ mice carrying mutant human SOD1 gene develop similar symptoms and pathological features as ALS [2], and this mouse model has been widely used in the field of ALS research.

Microglia play a dual role in the pathogenesis of ALS. On one hand, it has been reported that activated microglia contributed to MNs death in ALS mice via induction of inflammatory responses [3–6]. Inhibition of microglial activation could ameliorate disease pathologies through the blockage of NF-κB signaling in SOD1^G93A^ mice [7]. On the other hand, one recent study has found that microglia can promote the recovery of MNs function in a mouse model of TDP-43 proteinopathy [8]. The cytotoxic or neuroprotective roles of microglia may be dependent on their phenotypes, M1 and M2 [9]. M1 microglia is characterized by the elevated expression of inducible nitric oxide synthase (iNOS) and cluster of differentiation 86 (CD86), and can usually reduce neuronal survival through the overproduction of reactive oxygen species (ROS) and pro-inflammatory cytokines such as tumor necrosis factor α (TNF-α) and Interleukin 1β (IL-1β). M2 microglia is characterized by the expression of arginase 1 (Arg1) and cluster of differentiation 206 (CD206), which can enhance neuronal survival through the release of trophic and anti-inflammatory factors such as insulin-like growth factor 1(IGF-1) [10]. It has been speculated that M2 microglia may protect MNs through preventing neuroinflammation at an early stage, whereas M1 microglia induce MNs loss by promoting neuroinflammation during the late phase of disease [11].

Communications between MNs and microglia are thought to play an important role in the pathogenesis of ALS [12, 13]. While microglia regulate MNs fates, MNs are also able to influence microglial activity through various mechanisms [14, 15]. For example, extracellular mutant SOD1 protein, predominately secreted from MNs, directly triggers microglial activation [14, 16]. Chemokine (C-X3-C motif) ligand 1 (CX3CL1), also named fractalkine, is the only representative of the CX3C chemokine, being constitutively expressed by neurons. Strong neuronal excitation can cause fractalkine to be cleaved from the neuronal surface. This soluble form of fractalkine then interacts with nearby microglia which express low levels of CX3CL1 receptor CX3CR1 [17]. The dynamic changes in the expression of CX3CL1 in MNs and its receptor CX3CR1 in microglia may be involved in fundamental processes of neurons/microglia communication [18]. Disruption of CX3CL1/CX3CR1 axis could result in microglial activation and overexpression of IL-1β, TNFα [19]. Moreover, polymorphism of the CX3CR1 gene can influence the fate of ALS [20]. Until now, the dynamic changes of CX3CL1/CX3CR1 axis in the spinal cord anterior horn of ALS are not well-documented during disease progression. Therefore, in the present study, we aimed to examine the dynamic changes of CX3CL1/CX3CR1 axis and detected the microglial activation status and the related cytokines production in the anterior horn region of spinal cord in SOD1^G93A^ mouse model of ALS at the age of 40, 60, 90 and 120 days. The study outcome may help us understand the roles of CX3CL1/CX3CR1 axis in the disease progression of ALS and may provide new clues for ALS therapy.

## Materials and methods

### SOD1^G93A^ transgenic mice and their treatments

Male SOD1^G93A^ transgenic (TG) mice overexpressing 20 copies of mutant human SOD1 with a Gly93Ala substitution (SOD1^G93A^) were obtained from Jackson Laboratories (B6SJL-Tg(SOD1*G93A)1Gur/J, stock number 002726). The hemizygous TG mice start to show a decrease of max running speed and abnormal gait from 50 days of age. Around 90–100 days of age, visible symptoms of hind limb paralysis could be found, followed by a complete hind limb paralysis around 125 days of age [21]. All animals were housed under standard conditions of constant temperature and controlled lighting (12/12 h light/dark cycle). TG mice were maintained as heterozygote by crossing TG males with wild-type (WT) females of the same C57BL/6 J genetic background. At 30 days, genotypes of TG mice were identified by PCR according to our previously reported protocol [22]. In order to exclude the possible impacts of gender difference on disease onset and lifespan [23], only male TG and WT mice were used in the current study.

### Anterior horn tissue separation and RNA extraction

According to the pathological stages of ALS [24], male TG mice and their age-matched WT littermates were sacrificed at the age of 40 days (asymptomatic stage), 60 days (pre-symptomatic stage), 90 days (symptomatic stage) and 120 days (terminal stage). Then the mice were perfused through heart with ice-cold 0.1 M PBS. The lumbar spinal cord was separated on ice. The anterior horn region was prepared by cutting ventral half of the lumbar spinal cord and removing the tissues surrounding anterior horn that marked with the black line (as shown in Additional file 1: Figure S1) under the dissect microscope. The tissues were collected and stored at − 80 °C. Total RNA was extracted from the anterior horns and lysed in RNAiso plus reagent according to manufacturer’s instructions (Takara Bio, Japan). RNA concentration and purity were measured by UV spectroscopy.

### Complementary DNA (cDNA) synthesis and quantitative real-time PCR (qPCR)

cDNA was obtained by reverse transcription using the primescript RT reagent kit with gDNA eraser (Takara Bio, Japan) by two-step method. qPCR was performed with TransStart TOP green qPCR supermix (Transgen Biotech, China) using the Applied Biosystems 7500 Real-Time PCR System (Thermo Fisher Scientific, USA). GAPDH was selected as an internal control for gene level. The primers sequences for qPCR were summarized in Table 1. The relative level was calculated as 2^–ΔΔCt^ normalizing to GAPDH and relative to age-matched WT mice.

**Table 1:** Primers for qPCR

Primer	Forward primer	Reverse primer
GAPDH	TGTGTCCGTCGTGGATCTGA	TTGCTGTTGAAGTCGCAGGAG
CD206	TCAGCTATTGGACGCGAGGCA	TCCGGGTTGCAAGTTGCCGT
CD86	ACGATGGACCCCAGATGCACCA	GCGTCTCCACGGAAACAGCA
INOS	CCCTCCTGATCTTGTGTTGGA	CAACCCGAGCTCCTGGAA
IL-1β	TGCCACCTTTTGACAGTGATG	ATGTGCTGCTGCGAGAATTTG
TNF-α	CCAGTGTGGGAAGCTGTCTT	AAGCAAAAGAGGAGGCAACA
Arg1	CTTGCGAGACGTAGACCCTG	TCCATCACCTTGCCAATCCC
CX3CR1	CTGTTATTTGGGCGACATTG	AACAGATTTCCCACCAGACC
CX3CL1	ATTGGAAGACCTTGCTTTGG	GCCTCGGAAGTTGAGAGAGA
IL-10	GGCAGAGAAGCATGGCCCAGAA	AATCGATGACAGCGCCTCAGCC

### Frozen sections for immunofluorescent staining

TG mice at 40, 90 and 120 days and age-matched WT littermates were sacrificed by perfusion through heart with ice-cold 0.1 M PBS and 4% paraformaldehyde (PFA). The lumbar spinal cords were removed and immersed in 4% PFA. After 12 h, the tissues were gradually dehydrated in 15 and 30% sucrose solution for 24 h, then embedded in Optimal Cutting Temperature Compound and cut into serial sections of 10 μm thickness in freezing microtome at − 25 °C. Sections were attached to glass slides for immunofluorescent staining as previously described [22]. Spinal cord sections were baked for 30 min at 55 °C and washed by 0.1 M PBS for three times, then blocked with 5% bovine serum and 0.3% triton in PBS for 1 h at room temperature. Sections were incubated with following primary antibodies overnight at 4 °C: anti-NeuN (MAB377, 1:500, Millipore), anti-Iba1 (019–19,741, 1:1000, WAKO), anti-CX3CL1 (AF472, 1: 200, R&D Systems), anti-CX3CR1 (AF5825, 1: 50, R&D Systems), anti-CD86 (553,689, 1:1000, BD Biosciences), anti-Arg1 (sc-271,430, 1:50, Santa Cruz), anti-GFAP (Z0334, 1:2000, Dako). After washing in PBS three times, secondary donkey anti-rabbit IgG conjugated Alexa 555 (A0453, 1:2000, Beyotime), IFKine™ Green donkey anti-goat IgG (A24231, 1:500, Abbkine), goat anti-rabbit IgG conjugated Alexa 594 (8889S, 1:2000, Cell Signaling Technology), goat anti-mouse IgG conjugated Alexa 488 (4408S, 1:2000, Cell Signaling Technology), Cy3 goat anti-rat IgG (A0507, 1:2000, Beyotime, China), goat anti-mouse IgG conjugated Alexa 594 (8890S, 1:2000, Cell Signaling Technology) were applied respectively at room temperature for 2 h. Hoechst was used for nuclei staining. After washing, sections were covered with mounting medium and analyzed by fluorescent microscope.

### Cell counting

For CX3CR1^+^/Iba1^+^, CD86^+^ /Iba1^+^ or Arg1^+^/Iba1^+^ cell counting, five sections spaced 100 μm apart in each mouse were used and both sides of the anterior horn region (two views in one slide and at least five slides per animal) were captured at 200 × magnification. Then the total number of cells in both sides of anterior horn region was manually counted by a person who was blind to experimental design. Counts from both sides of anterior horn region were averaged and divided by the area in square millimeters. The average count from five sections was used as a single measurement per mouse for statistical analysis. The integrated density of CX3CL1 staining was measured by Image J software on all five sections per animal [25].

### Western blotting and qualification

Anterior horns in lumbar spinal cords from both TG mice and WT littermates were dissected and sonicated in ice cold lysis buffer (P1003B, Beyotime, China). The total protein was extracted and separated by 10% SDS-polyacrylamide gel electrophoresis and then transferred onto nitrocellulose membranes (Merck Millipore, Germany). After blocking with 5% skim milk prepared in tris-buffered saline-Tween 20 (TBST) (20 mM Tris, pH 7.2, 150 mM NaCl, 0.1% Tween 20) for 1 h at room temperature, membranes were incubated for 14 h at 4 °C with primary antibodies against CX3CL1 (bs-0811R, 1: 1000, Bioss), CX3CR1 (AF5825, 1: 100, R&D Systems), β-tubulin (2128S, 1:2000, Cell Signaling Technology). After washing, membranes were incubated with HRP-conjugated goat-anti-rabbit IgG (7074S, 1:2000, Cell Signaling Technology) or HRP-conjugated donkey-anti-goat IgG antibody (A0181, 1:2000, Beyotime) for 1 h at room temperature. The target protein bands were quantified by using FluorChem Q system (ProteinSimple, San Jose, CA, USA).

### Statistical analysis

Data were expressed as Mean ± Standard deviation (SD). Statistical analysis was performed using statistical program for social sciences (SPSS) 22.0. Two-way ANOVA with Tukey post-hoc for multiple comparisons was used to determine whether significant differences existed between TG and WT mice at different time points. A *p* value of less than 0.05 indicates statistical significance.

## Results

### CX3CL1 was highly expressed in MNs and decreased during disease progression in TG mice

**Fig. 1:**
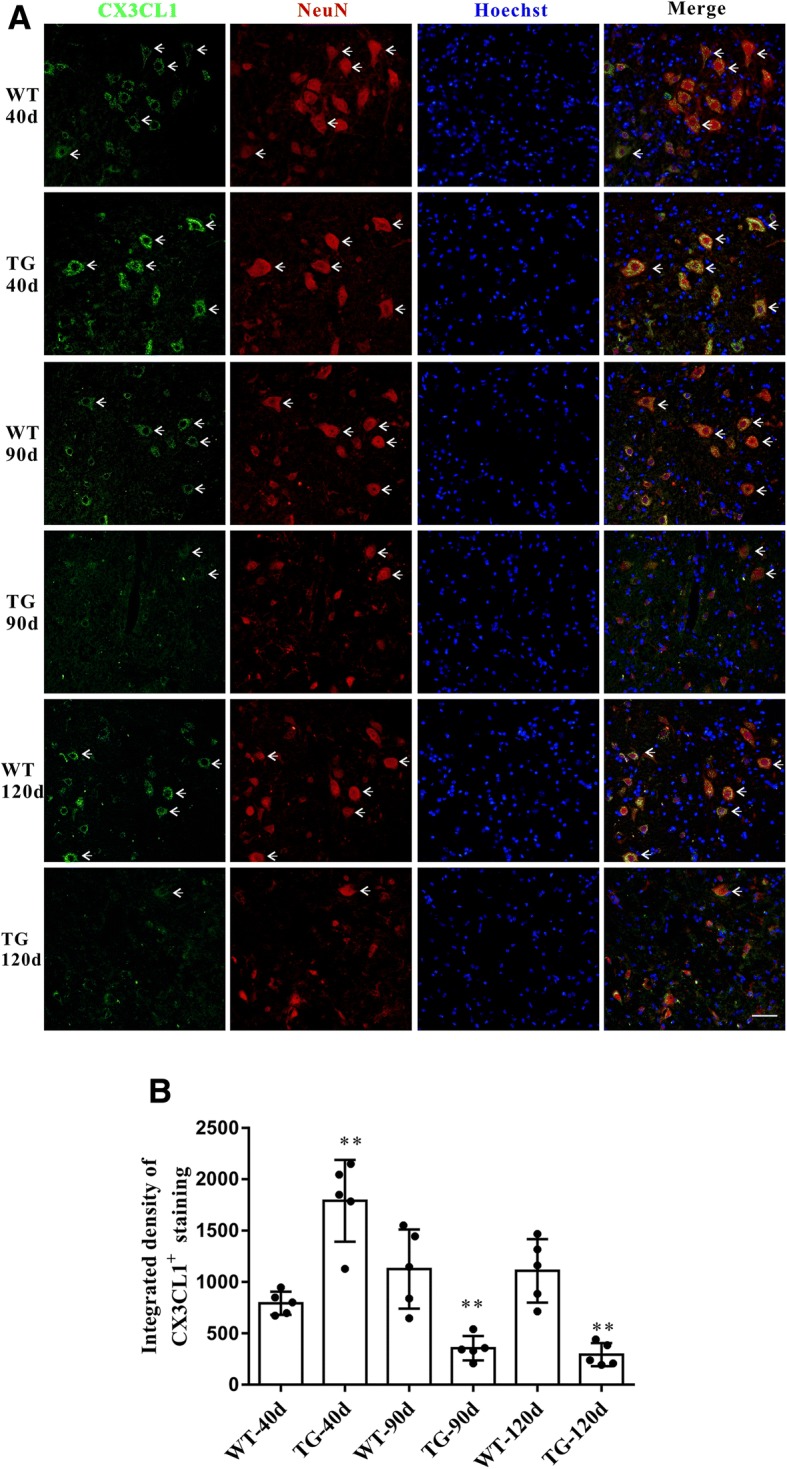
CX3CL1 were mainly located in neurons and decreased during MNs loss in TG mice. **a** Double immunofluorescent staining of CX3CL1 (green) and NeuN (red) in the anterior horn of lumber spinal cord of WT and TG mice at the age of 40, 90 and 120 days. Scale bar = 50 μm. **b** Integrated density of CX3CL1 staining. The values are expressed as mean ± SD, *p < 0.05 versus age-matched WT mice, ** *p* < 0.001 versus age-matched WT mice, *n* = 5 in each group

Dramatic MNs loss in the spinal cord anterior horn region is an important feature of ALS mice during disease progression [24]. The immunoflurescent staining showed that CX3CL1 was mainly expressed in MNs (Fig. 1a), and no obvious CX3CL1 staining was observed on microglia or astrocytes of ALS mice at the age of 90 days (Additional file 2: Figure S2). Interestingly, the integrated density of CX3CL1 staining in MNs was elevated in the spinal cord anterior horn region of TG mice at 40 days but decreased accompanied with the MNs loss in the anterior horn region at the age of 90 and 120 days (Fig. 1b).

**Fig. 2:**
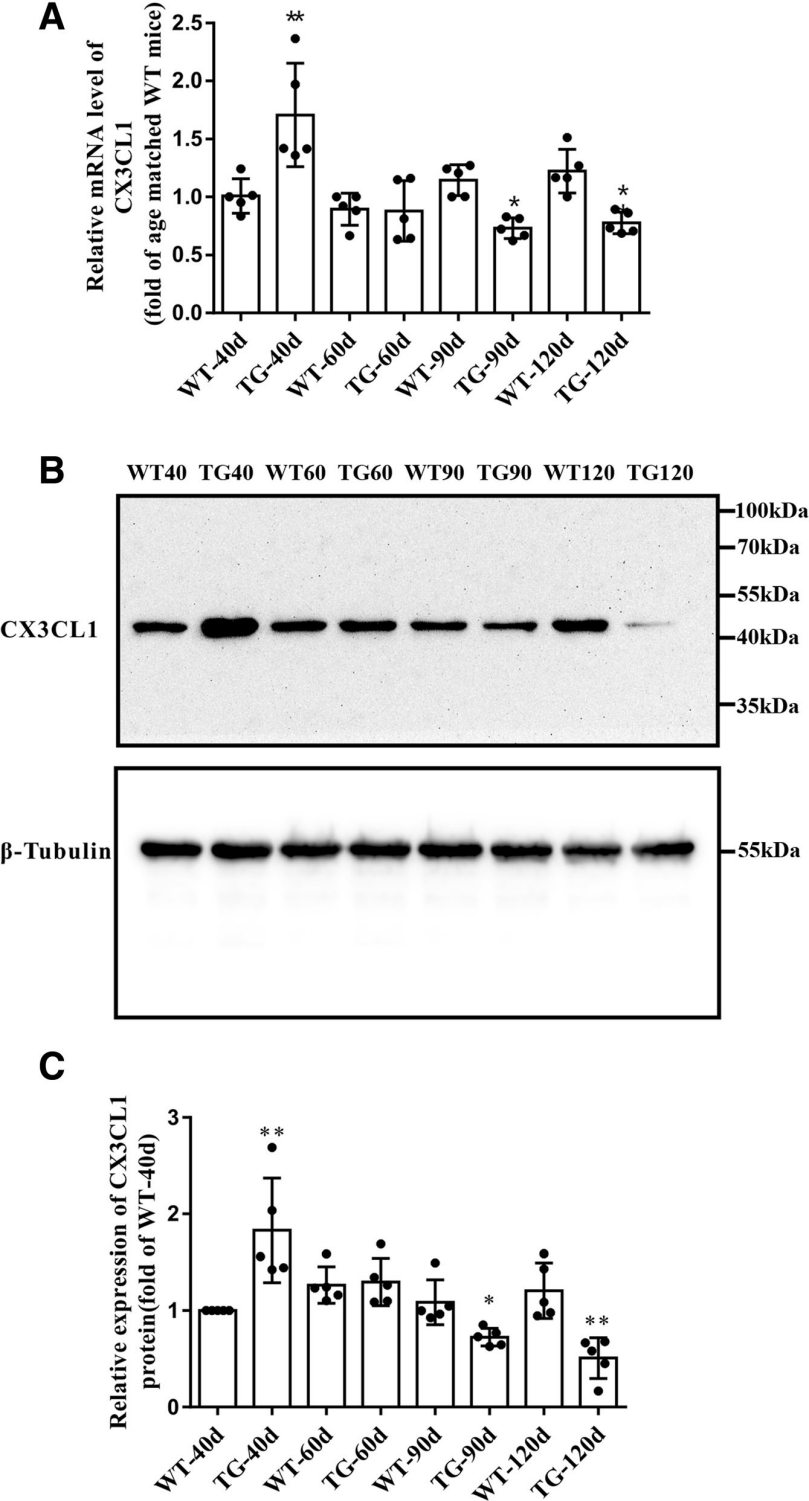
The mRNA and protein levels of CX3CL1 in the spinal cord anterior horns. **a** qPCR analysis of CX3CL1 mRNA level. **b** Western blot analysis of CX3CL1 protein level. **c** Quantitative analysis the CX3CL1 Protein level. The values are expressed as mean ± SD, *p < 0.05 versus age-matched WT mice, ** *p* < 0.001 versus age-matched WT mice, *n* = 5 in each group

We further measured the mRNA level of CX3CL1. At 40 days of age, the mRNA level of CX3CL1 in the spinal cord anterior horn region of TG mice was 1.7 fold higher than that of WT mice; however, at 90 and 120 days, the mRNA level of CX3CL1 in the spinal cord anterior horn region of TG mice was significantly lower than that of WT mice (Fig. 2a). Western blot also revealed similar dynamic change of CX3CL1 protein, as shown by the elevated level of CX3CL1 in TG mice at 40 days but declined at 90 and 120 days, as compared with age-matched WT mice (Fig. 2b, c). All these data may predict a loss of CX3CL1 signaling in MNs during the pathogenesis and progression of ALS.

### CX3CR1 was mostly located in microglia and consistently increased in TG mice at 90 and 120 days

**Fig. 3:**
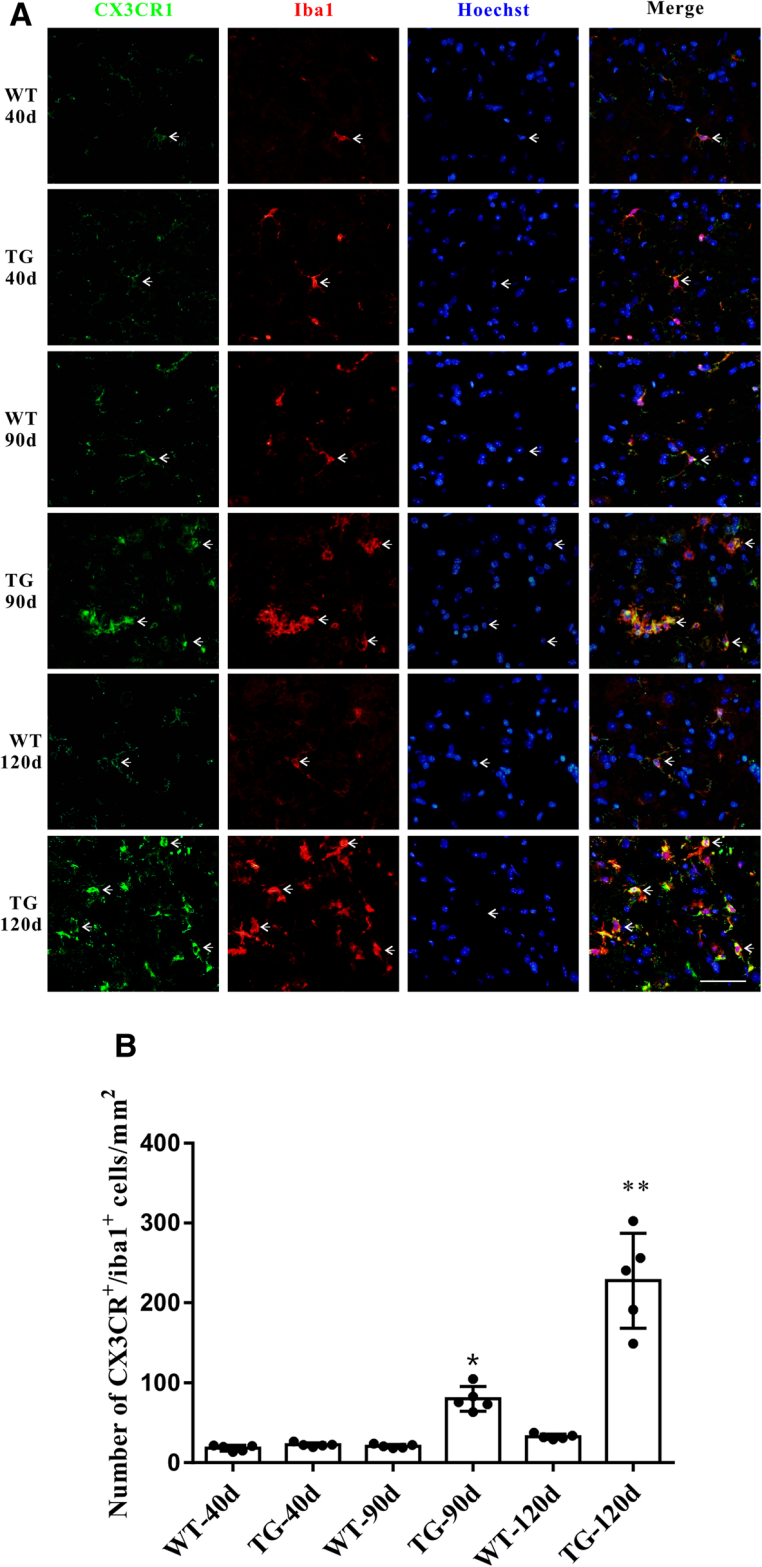
CX3CR1 was mainly located in microglia and was increased in the TG mice at the age of 90 and 120 days. **a** Double immunofluorescent staining of CX3CR1 (green) and Iba1(red) in the anterior horn of lumber spinal cord of WT and TG mice at the age of 40, 90 and 120 days. Scale bar = 50 μm. **b** Number of CX3CR1^+^/Iba1^+^ microglia per mm^2^. The values are expressed as mean ± SD, *p < 0.05 versus age-matched WT mice, ** *p* < 0.001 versus age-matched WT mice, *n* = 5 in each group

**Fig. 4:**
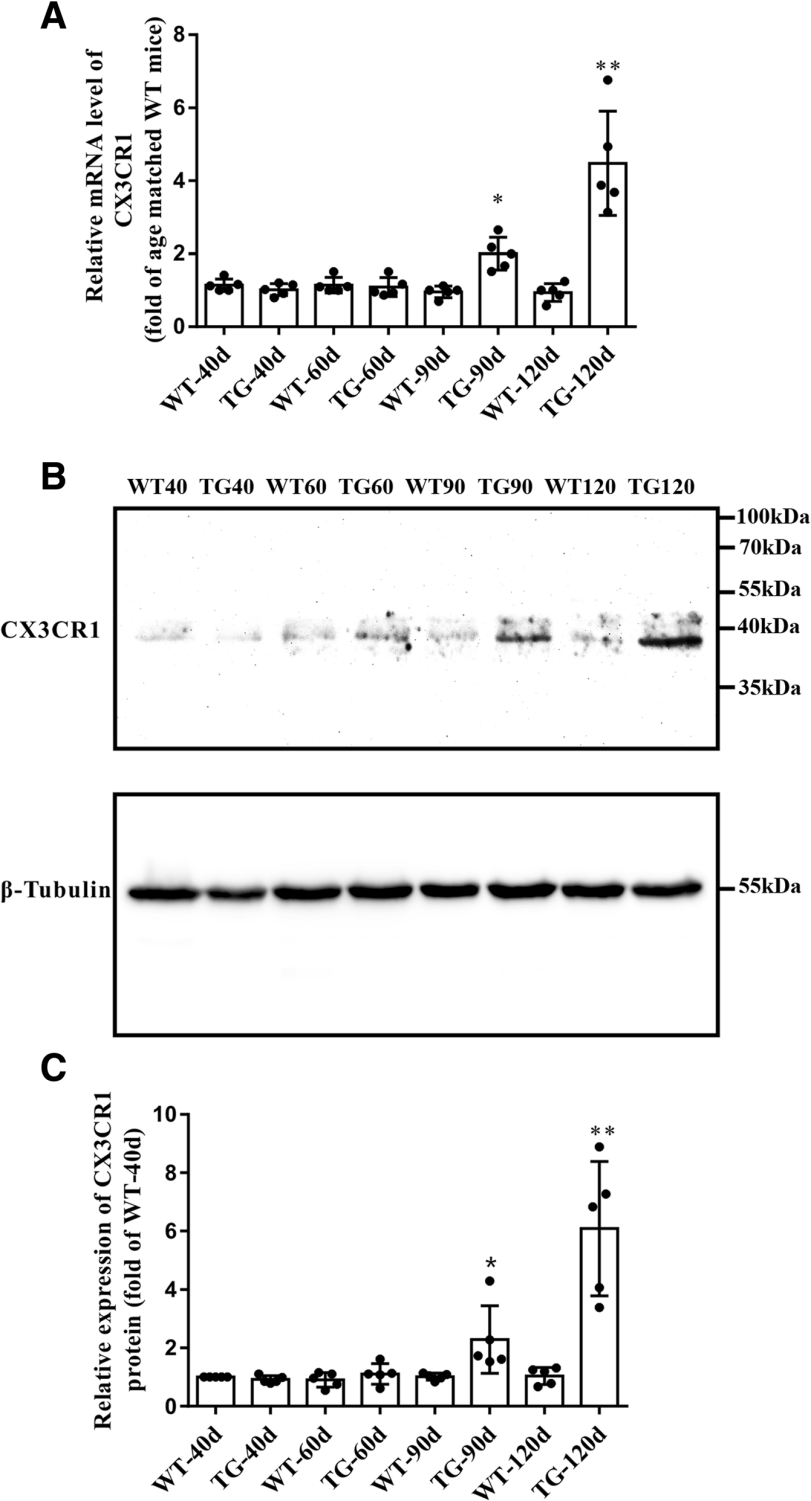
The mRNA and protein levels of CX3CR1 in the spinal cord anterior horns. **a** qPCR analysis of CX3CR1 mRNA level. **b** Western blot analysis of CX3CR1 protein level. **c** Quantitative analysis the CX3CR1 Protein level. The values are expressed as mean ± SD, *p < 0.05 versus age-matched WT mice, ** *p* < 0.001 versus age-matched WT mice, *n* = 5 in each group

As the disease progression, significant microgliosis and microglial activation could be found in the spinal cord anterior horn region of TG mice [26]. We found that the CX3CL1 receptor CX3CR1 was dominantly located in microglia and its expression was consistently increased together with microglial proliferation (Fig. 3a, b). Moreover, compared with WT mice, CX3CR1 mRNA level did not increase as CX3CL1 did in the spinal cord anterior horn region of TG mice at 40 days, but increased significantly at 90 and 120 days (Fig. 4a). Western blot also revealed a consistent elevation of CX3CR1 protein in the spinal cord anterior horn region of TG mice at 90 and 120 days compared with their age-matched WT mice (Fig. 4b, c). The dynamic changes of CX3CR1 might be a consequence of direct microgliosis or a compensational response to CX3CL1 downregulation.

### Imbalanced M1 and M2 microglial activation in the spinal cord anterior horn region of TG mice at 120 days

**Fig. 5:**
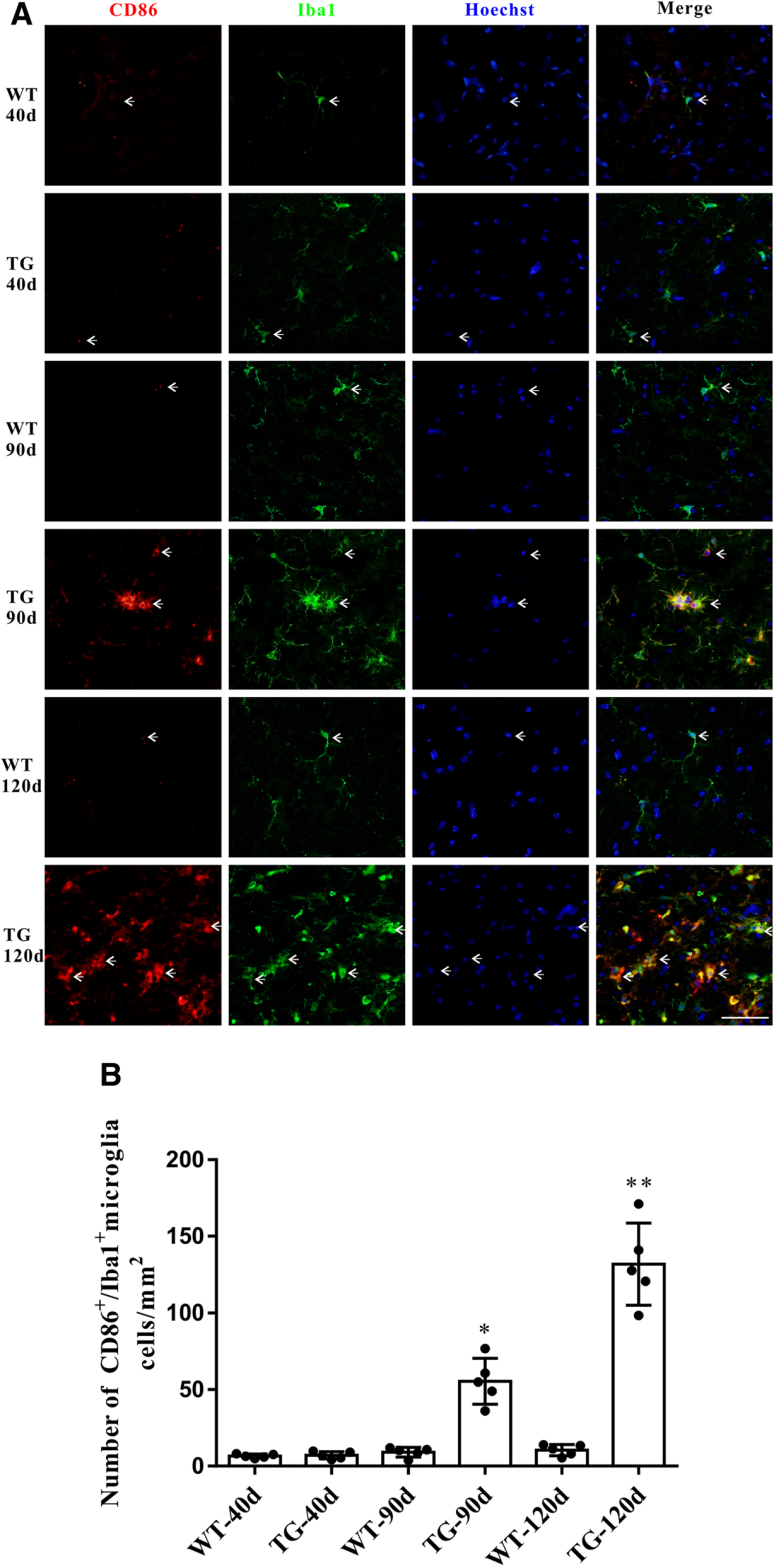
The changes of M1 microglia in the TG mice at different time points. **a** Double immunofluorescent staining of CD86 (red) and Iba1 (green) at different time points, Sale bar = 50 μm. **b** Quantitative analysis of the M1 microglia per mm^2^ at different time points. The values are expressed as mean ± SD, *p < 0.05 versus age-matched WT mice, ** *p* < 0.001 versus age-matched WT mice, *n* = 5 in each group

**Fig. 6:**
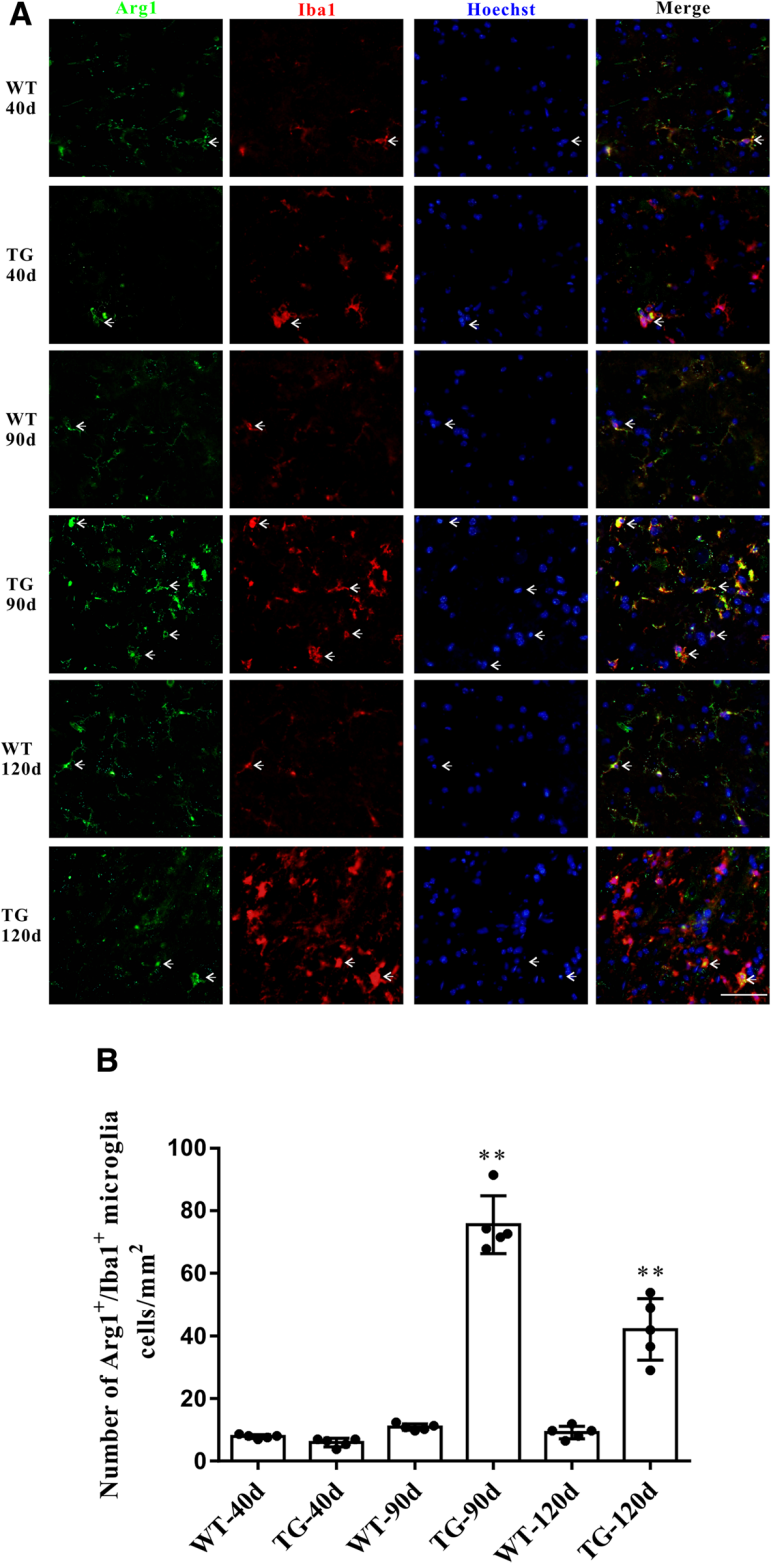
The changes of M2 microglial phenotypes in TG mice at different time points. **a** Double immunofluorescent staining of Arg1 (green) and Iba1 (red) at different time points, Sale bar = 50 μm. **b** Quantitative analysis of the M2 microglia per mm^2^ at different time points. The values are expressed as mean ± SD, *p < 0.05 versus age-matched WT mice, ** *p* < 0.001 versus age-matched WT mice, *n* = 5 in each group

**Fig. 7:**
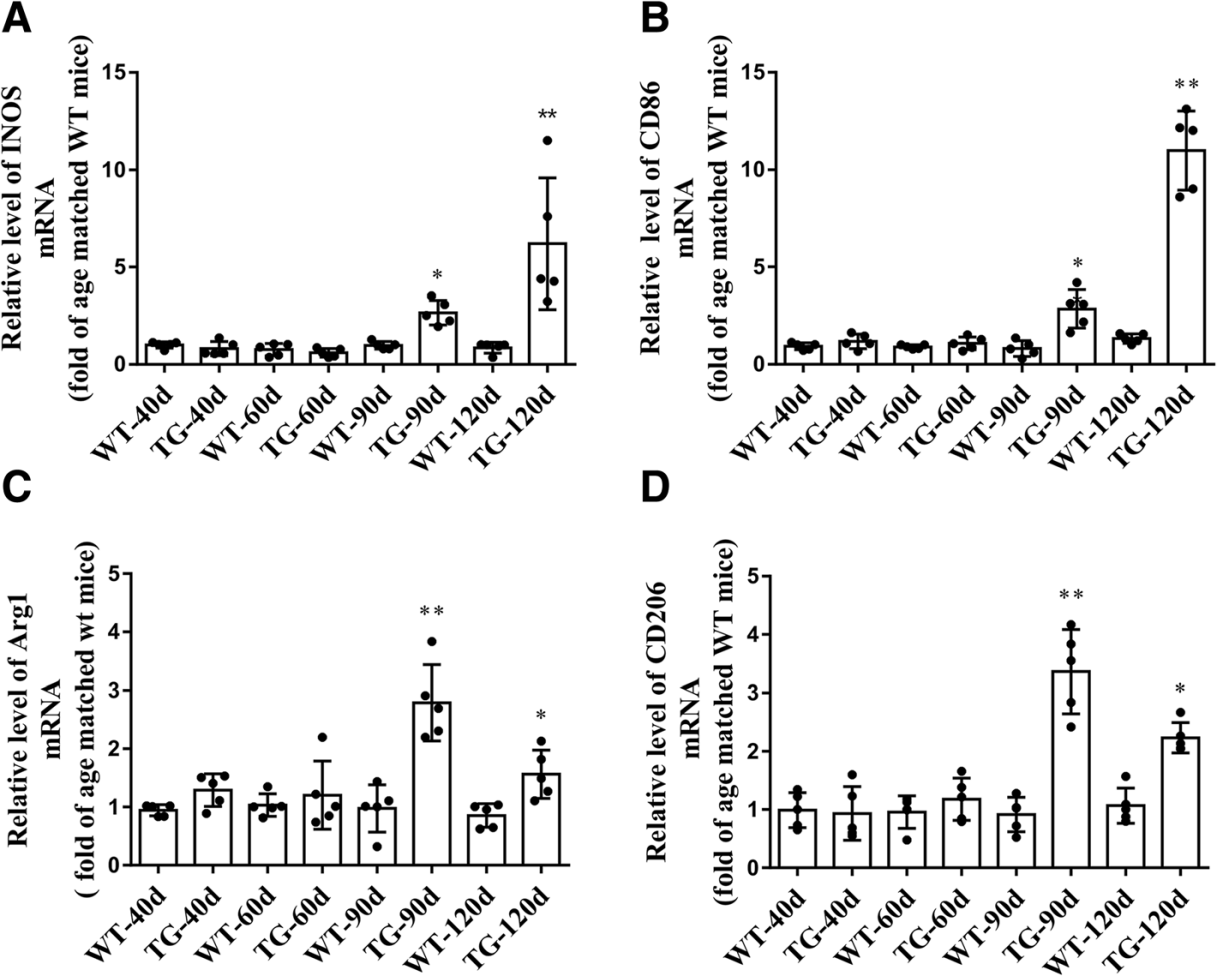
The mRNA levels of M1/M2 microglial markers in the TG mice at different time points. **a**, **b** The mRNA levels of M1 markers (INOS, CD86). **c**, **d** The mRNA levels of M2 markers (Arg1, CD206). The values are expressed as mean ± SD, *p < 0.05 versus age-matched WT mice, ** *p* < 0.001 versus age-matched WT mice, *n* = 5 in each group

Accompanied by the impaired CX3CL1/CX3CR1 axis, the number of activated microglia was dramatically increased in the spinal cord anterior horn region of TG mice, evidenced by the immunoflurescent changes of M1/M2 microglia markers. The number of CD86^+^/Iba1^+^ M1 microglia was increased at 90 days and further increased at 120 days (Fig. 5a and b), whereas the number of Arg1^+^/Iba1^+^ M2 microglia was significantly increased at 90 days but declined in TG mice at 120 days (Fig. 6a and b). These results were further confirmed by qPCR, as shown by the increased mRNA levels (about 2.7–3.4 fold) of both M1 phenotype (CD86, iNOS) and M2 phenotype (CD206, Arg1) microglial markers in the spinal cord anterior horn region of TG mice at 90 days. Moreover, consistent with the microglial quantification analysis, at the age of 120 days, while M1 microglial markers (CD86, INOS) were persistently increased (about 6.2–11.0 fold higher than those in age-matched WT mice), mRNA levels of M2 microglial markers (CD206, Arg1) were reduced compared with those at 90 days of age (still significantly higher than those in WT mice) (Fig. 7). All these data may predict an altered M1/M2 polarization during ALS progression, as indicated by an activated M1/M2 microglia at 90 days and subsequently a relatively suppressed M2 polarization at 120 days.

**Fig. 8:**
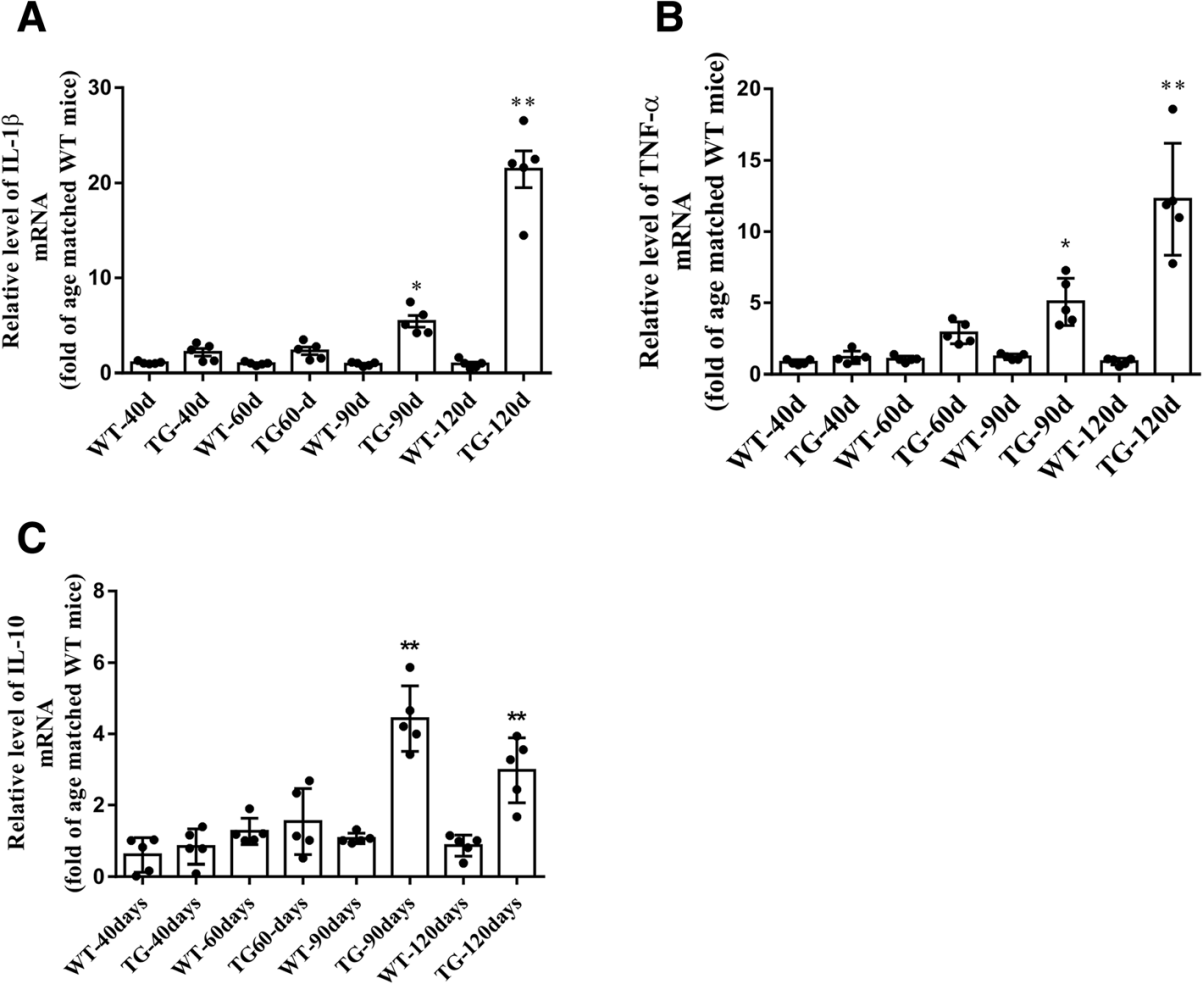
The changes of M1/M2 microglia related cytokines mRNA levels at different time points. **a**, **b** mRNA levels of two cytokines related to M1 microglia (IL-1β and TNF-α). **c** mRNA levels of IL-10 related to M2 microglia. The values are expressed as mean ± SD, *p < 0.05 versus age-matched WT mice, ** *p* < 0.001 versus age-matched WT mice, *n* = 5 in each group

Microglia play important roles in modulating neuroinflammation through secreting proinflammatory or anti-inflammatory cytokines. As proinflammatory and neuro-toxic cytokines, IL-1β and TNF-α are secreted by M1 phenotype microglia and cause neuronal cell death in most neurodegenerative disease. In contrast, as a neuro-protective cytokine associated with M2 phenotype microglia, interleukin-10 (IL-10) can inhibit the production of neuro-toxic factors by microglia to promote cell survival. Here, we found that the mRNA levels of IL-1β and TNF-α were increased by about 5.1–5.4 fold in spinal cord of TG mice at 90 days, and persistently increased by about 12.3–21.5 fold at 120 days, compared with age-matched WT mice (Fig. 8a, b). Moreover, the IL-10 mRNA increased by about 4.4 fold at 90 days but reduced to about 3.0 fold at 120 days in TG mice compared with age matched WT mice (Fig. 8c). These results were consistent with the data of microglial quantification and microglial markers determination and further indicate the imbalanced activation of M1/M2 microglia in the spinal cord anterior horn region of TG mice at 120 days.

## Discussion

Microglia have been studied extensively for their roles in various neurodegenerative diseases including ALS. Their activation has even been considered as the “hallmark” of many neurodegenerative conditions. However, the exact roles of microglia in ALS are still under debate. Previous study reported that lower mutant SOD1 expression within microglia significantly extended the survival of LoxSOD1^G37R^ mice [27], whereas a later study showed that the elimination of proliferating microglia couldn’t prevent MNs loss in SOD1^G93A^ mice [28]. A latest research revealed that microglia could selectively clear neuronal hTDP-43, which was necessary for recovery of MNs function in a mouse model of TDP-43 proteinopathy [8]. Therefore, manipulating the function of microglia has the potential to improve ALS prognosis.

As the only member of the CXC subfamily of chemokines, CX3CL1 exists in two distinct forms: a full-length membrane-bound form and a shed form containing the N-terminal chemokine domain. The shed chemokine domain of CX3CL1 acts, when cleaved, as a signaling molecule and binds to microglial-expressed CX3CR1 receptor to modulate microglial activation [18]. Here, we found CX3CL1 was up-regulated in the MNs at early stage of ALS (40 days) but decreased at the later phase of disease (90 and 120 days) in ALS model*.* Simultaneously, the expression level of CX3CR1, the microglia-located sole receptor for CX3CL1, was increased at the age of 90 and 120 days, consistent with the alteration of CX3CL1. In addition, accompanied with this impaired CX3CL1/CX3CR1 axis, microglia were consistently activated in the spinal cord anterior horn of ALS mice in a M1/M2 imbalanced manner.

Previous studies have shown that the impaired CX3CL1–CX3CR1 axis was usually accompanied by abnormal microglial activation in various animal models of neurological diseases [29–31]. It has been reported that CX3CL1 could attenuate LPS-induced microglial activation by inducing phosphatidylinositol-3 kinase pathway and reducing the production of nitric oxide (NO), IL-6, and TNF-α [32]. Microglial activation usually occurred when CX3CL1 was decreased [32, 33]. Moreover, CX3CL1 protein could be up-regulated in injured neurons to protect neurons against glutamate excitotoxicity and cell death [34, 35]. The deficiency of its receptor CX3CR1 could promote microglial activation and exacerbate MNs loss in ALS mice [36]. Here, we found a significant increase of CX3CL1 level in MNs of the anterior horn of ALS mice at the age of 40 days, while the TG mice showed neither obvious clinical symptoms nor MNs loss. We assumed that the increased CX3CL1 level might be the early sign of MNs degeneration, which may in turn affect the microglial function at the early stage of disease. After disease onset, there was a significant decrease of CX3CL1 immunoflurescent staining on remaining MNs in TG mice. It is likely that the CX3CL1 reduction after disease onset may reflect the abnormally reduced expression on MNs and MNs loss as well.

Microglial proliferation may result in CX3CR1 elevation at 90 and 120 days. Up-regulation of CX3CR1 may be also a compensation for CX3CL1 down-regulation in the spinal cord of ALS model. The function of CX3CL1/CX3CR1 axis requires the coordination between CX3CL1 and CX3CR1 level, and the change of CX3CR1 alone may not be able to compensate for the function of CX3CL1/CX3CR1 axis. The impaired CX3CL1/CX3CR1 axis seems to be associated with microglial activation at ALS disease onset and might contribute to the disease progression. Further study on the molecular mechanisms of CX3CL1/CX3CR1 axis in regulating microglial activation might provide useful information to determine the value of CX3CL1/CX3CR1 axis as a target for ALS therapy.

Moreover, in the present study, we also found an altered microglial polarization during disease progression of ALS. Microglia are the resident immune cells of the central nervous system (CNS) and are the primary mediators of neuroinflammation. The activated microglia have been categorized into the classical M1 and alternative M2 phenotype based on their polarization status [9]. Activation of microglia is regulated by multiple extracellular and intracellular factors, cross talk between neurons and microglia could modulate M1 or M2 microglial activation during progression of ALS [37, 38]. It is believed that M2 polarization may be triggered as a protective compensation during the early stage of ALS, while a strong M1 polarization may be dominant during the later phases of the disease in ALS [38]. Adult microglia isolated from ALS mice at disease onset showed an M2 phenotype and protected MNs, whereas microglia isolated from ALS mice in end-stage of disease adopted a neurotoxic M1 phenotype [39]. Our present data revealed that the dynamic changes of CX3CL1/CX3CR1 axis might be associated with alteration of microglial phenotype as ALS progression in the TG mice. CX3CR1 could regulate microglial phenotypes in a time dependent manner. During the early disease stage CX3CR1 may be a protective molecule. However, as disease progresses, it becomes harmful to neurons [40]. Although M1 microglia and pro-inflammatory cytokines were significantly increased at the age of 90 days, the increased M2 microglia and anti-inflammatory cytokines were paralleled with elevated M1 microglia. Thus, this balance between M1 and M2 microglial polarization may be result in stabilization of ALS at initial disease stage. Following the disease progression, the role of CX3CR1 is changed, and the balance between M1 and M2 microglia might be disrupted, as shown by a continuous increase in M1 microglia polarization and overproduction of pro-inflammatory cytokines, but a declined M2 microglia activation and decreased anti-inflammatory cytokines production. These results indicate that an altered M1/M2 microglial activation may contribute to the ALS progression, and reversing such imbalance between M1 and M2 microglia may delay the progression of ALS.

## Conclusions

In conclusion, impaired CX3CL1/CX3CR1 axis and imbalanced M1/M2 microglial activation may play an important role in the pathogenesis and progression of MNs degeneration in ALS. Regulating CX3CL1/CX3CR1 axis and microglial activation may be a feasible option for future ALS therapy.

## Additional files

Additional file 1: **Figure S1.** Roadmap for the dissection of anterior horn region in lumber spinal cord. (TIF 1640 kb)

Additional file 2: **Figure S2.** The immunofluorescent staining of CX3CL1 on microglia or astrocytes. (A) Double immunofluorescent staining of CX3CL1 (green) and GFAP (red) positive astrocytes. (B) Double immunofluorescent staining of CX3CL1 (green) and Iba1 (red) positive microglia. Scale bar = 50 μm. (TIF 1941 kb)

## Abbreviations

ALS: Amyotrophic lateral sclerosis
Arg1: Arginase 1 (Arg1)
CCL: CC chemokine ligands
CD206: Cluster of differentiation 206
CD86: Cluster of differentiation 86
CNS: Central nervous system
CX3CL1: Chemokine (C-X3-C motif) ligand 1
CX3CR1: Chemokine (C-X3-C motif) ligand 1 receptor
IL-10: Interleukin-10
IL-1β: Including interleukin-1β
IL-4: Interleukin-4
iNOS: Inducible nitric oxide synthase.
MNs: Motor neurons
SOD1: Cu/Zn superoxide dismutase 1
TGF-β: Transforming growth factor-β
TNF-α: Tumor necrosis factor-α

## References

[1] Ferraiuolo L, Kirby J, Grierson AJ, Sendtner M, Shaw PJ (2011). Molecular pathways of motor neuron injury in amyotrophic lateral sclerosis. Nat Rev Neurol.

[2] Gurney ME, Pu H, Chiu AY, Dal Canto MC, Polchow CY, Alexander DD (1994). Motor neuron degeneration in mice that express a human Cu, Zn superoxide dismutase mutation. Science.

[3] Clement AM, Nguyen MD, Roberts EA, Garcia ML, Boillee S, Rule M (2003). Wild-type nonneuronal cells extend survival of SOD1 mutant motor neurons in ALS mice. Science.

[4] Pramatarova A, Laganière J, Roussel J, Brisebois K, Rouleau GA (2001). Neuron-specific expression of mutant superoxide dismutase 1 in transgenic mice does not lead to motor impairment. J Neurosci.

[5] Lino MM, Schneider C, Caroni P (2002). Accumulation of SOD1 mutants in postnatal motoneurons does not cause motoneuron pathology or motoneuron disease. J Neurosci.

[6] Puentes F, Malaspina A, van Noort JM, Amor S (2016). Non-neuronal cells in ALS: role of glial, immune cells and blood-CNS barriers. Brain Pathol.

[7] Frakes AE, Ferraiuolo L, Haidet-Phillips AM, Schmelzer L, Braun L, Miranda CJ (2014). Microglia induce motor neuron death via the classical NF-kappaB pathway in amyotrophic lateral sclerosis. Neuron.

[8] Spiller KJ, Restrepo CR, Khan T, Dominique MA, Fang TC, Canter RG (2018). Microglia-mediated recovery from ALS-relevant motor neuron degeneration in a mouse model of TDP-43 proteinopathy. Nat Neurosci.

[9] Tang Y, Le W (2016). Differential roles of M1 and M2 microglia in neurodegenerative diseases. Mol Neurobiol.

[10] Henkel JS, Beers DR, Zhao W, Appel SH (2009). Microglia in ALS: the good, the bad, and the resting. J Neuroimmune Pharmacol.

[11] Zhao W, Beers DR, Appel SH (2013). Immune-mediated mechanisms in the pathoprogression of amyotrophic lateral sclerosis. J NeuroImmune Pharmacol.

[12] Appel SH, Zhao W, Beers D, Henkel J (2011). The microglial-motoneuron dialogue in ALS. Acta Myol.

[13] Brites D, Vaz AR (2014). Microglia centered pathogenesis in ALS: insights in cell interconnectivity. Front Cell Neurosci.

[14] Urushitani M, Sik A, Sakurai T, Nukina N, Takahashi R, Julien JP (2006). Chromogranin-mediated secretion of mutant superoxide dismutase proteins linked to amyotrophic lateral sclerosis. Nat Neurosci.

[15] Biber K, Neumann H, Inoue K, Boddeke HW (2007). Neuronal ‘On’ and ‘Off’ signals control microglia. Trends Neurosci.

[16] Hernangomez M, Carrillo-Salinas FJ, Mecha M, Correa F, Mestre L, Loria F (2014). Brain innate immunity in the regulation of neuroinflammation: therapeutic strategies by modulating CD200-CD200R interaction involve the cannabinoid system. Curr Pharm Des.

[17] Chapman GA, Moores KE, Gohil J, Berkhout TA, Patel L, Green P (2000). The role of fractalkine in the recruitment of monocytes to the endothelium. Eur J Pharmacol.

[18] Harrison JK, Jiang Y, Chen S, Xia Y, Maciejewski D, McNamara RK (1998). Role for neuronally derived fractalkine in mediating interactions between neurons and CX3CR1-expressing microglia. Proc Natl Acad Sci U S A.

[19] Rogers JT, Morganti JM, Bachstetter AD, Hudson CE, Peters MM, Grimmig BA (2011). CX3CR1 deficiency leads to impairment of hippocampal cognitive function and synaptic plasticity. J Neurosci.

[20] Calvo A, Moglia C, Canosa A, Cammarosano S, Ilardi A, Bertuzzo D (2018). Commonpolymorphisms of chemokine (C-X3-C motif) receptor 1 gene modify amyotrophic lateral sclerosis outcome: A population-based study. Muscle Nerve.

[21] Kanning KC, Kaplan A, Henderson CE (2010). Motor neuron diversity in development and disease. Annu Rev Neurosci.

[22] Zhang X, Chen S, Song L, Tang Y, Shen Y, Jia L (2014). MTOR-independent, autophagic enhancer trehalose prolongs motor neuron survival and ameliorates the autophagic flux defect in a mouse model of amyotrophic lateral sclerosis. Autophagy.

[23] Choi CI, Lee YD, Gwag BJ, Cho SI, Kim SS, Suh-Kim H (2008). Effects of estrogen on lifespan and motor functions in female hSOD1 G93A transgenic mice. J Neurol Sci.

[24] Zhang X, Chen S, Li L, Wang Q, Le W (2010). Decreased level of 5-methyltetrahydrofolate: a potential biomarker for pre-symptomatic amyotrophic lateral sclerosis. J Neurol Sci.

[25] Schneider CA, Rasband WS, Eliceiri KW (2012). NIH Image to ImageJ: 25 years of image analysis. Nat Methods.

[26] Hall ED, Oostveen JA, Gurney ME (1998). Relationship of microglial and astrocytic activation to disease onset and progression in a transgenic model of familial ALS. Glia.

[27] Boillée S, Yamanaka K, Lobsiger CS, Copeland NG, Jenkins NA, Kassiotis G (2006). Onset and progression in inherited ALS determined by motor neurons and microglia. Science.

[28] Gowing G, Philips T, Van Wijmeersch B, Audet JN, Dewil M, Van Den Bosch L (2008). Ablation of proliferating microglia does not affect motor neuron degeneration in amyotrophic lateral sclerosis caused by mutant superoxide dismutase. J Neurosci.

[29] Castro-Sanchez S, Garcia-Yague AJ, Lopez-Royo T, Casarejos M, Lanciego JL, Lastres-Becker I. Cx3cr1-deficiency exacerbates alpha-synuclein-A53T induced neuroinflammation and neurodegeneration in a mouse model of Parkinson’s disease. Glia. 2018; 66(8):1752–62 10.1002/glia.23338 29624735

[30] Tang Z, Gan Y, Liu Q, Yin JX, Liu Q, Shi J (2014). CX3CR1 deficiency suppresses activation and neurotoxicity of microglia/macrophage in experimental ischemic stroke. J Neuroinflammation.

[31] Zhang L, Xu J, Gao J, Wu Y, Yin M, Zhao W. CD200-, CX3CL1-, and TREM2-mediated neuron-microglia interactions and their involvements in Alzheimer’s disease. Rev Neurosci. 2018; 29(8):837–48 10.1515/revneuro-2017-0084 29729150

[32] Lyons A, Lynch AM, Downer EJ, Hanley R, O’Sullivan JB, Smith A (2009). Fractalkine-induced activation of the phosphatidylinositol-3 kinase pathway attentuates microglial activation in vivo and in vitro. J Neurochem.

[33] Mecca C, Giambanco I, Donato R, Arcuri C. Microglia and aging: the role of the TREM2-DAP12 and CX3CL1-CX3CR1 axes. Int J Mol Sci. 2018;19(1):e318. 10.3390/ijms19010318 PMC5796261 29361745

[34] Chapman GA, Moores K, Harrison D, Campbell CA, Stewart BR, Strijbos PJ (2000). Fractalkine cleavage from neuronal membranes represents an acute event in the inflammatory response to excitotoxic brain damage. J Neurosci.

[35] Cerbai F, Lana D, Nosi D, Petkova-Kirova P, Zecchi S, Brothers HM (2012). The neuron-astrocyte-microglia triad in normal brain ageing and in a model of neuroinflammation in the rat hippocampus. PLoS One.

[36] Cardona AE, Pioro EP, Sasse ME, Kostenko V, Cardona SM, Dijkstra IM (2006). Control of microglial neurotoxicity by the fractalkine receptor. Nat Neurosci.

[37] Geloso MC, Corvino V, Marchese E, Serrano A, Michetti F, D’Ambrosi N (2017). The dual role of microglia in ALS: mechanisms and therapeutic approaches. Front Aging Neurosci.

[38] Hooten KG, Beers DR, Zhao W, Appel SH (2015). Protective and toxic neuroinflammation in amyotrophic lateral sclerosis. Neurotherapeutics.

[39] Liao B, Zhao W, Beers DR, Henkel JS, Appel SH (2012). Transformation from a neuroprotective to a neurotoxic microglial phenotype in a mouse model of ALS. Exp Neurol.

[40] Febinger HY, Thomasy HE, Pavlova MN, Ringgold KM, Barf PR, George AM (2015). Time-dependent effects of CX3CR1 in a mouse model of mild traumatic brain injury. J Neuroinflammation.

