# Supplementary figures and images for: Dynamic changes of CX3CL1/CX3CR1 axis during microglial activation and motor neuron loss in the spinal cord of ALS mouse model
Source: Transl Neurodegener. 2018 Dec 21;7:35. doi: 10.1186/s40035-018-0138-4 (PMC6309063; doi:10.1186/s40035-018-0138-4)

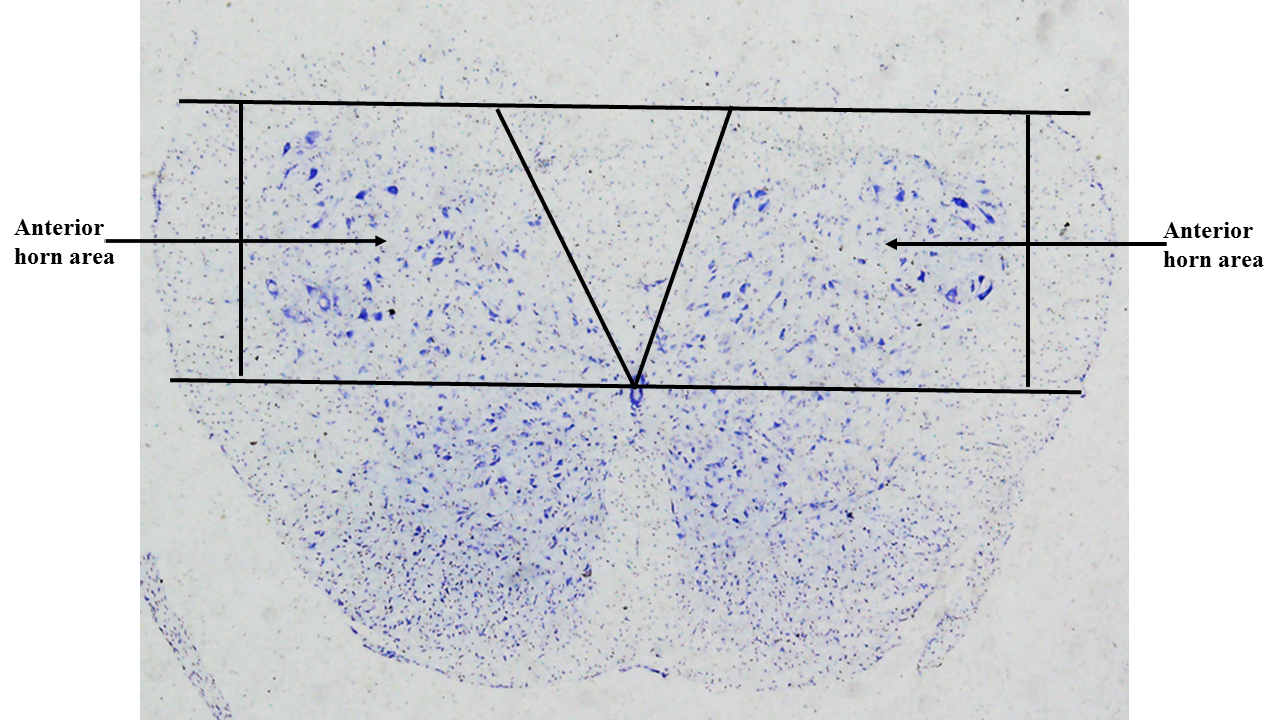

Supplement: Supplementary file 1 — Figure S1. Roadmap for the dissection of anterior horn region in lumber spinal cord. (TIF 1640 kb) [file 40035_2018_138_MOESM1_ESM.tif]

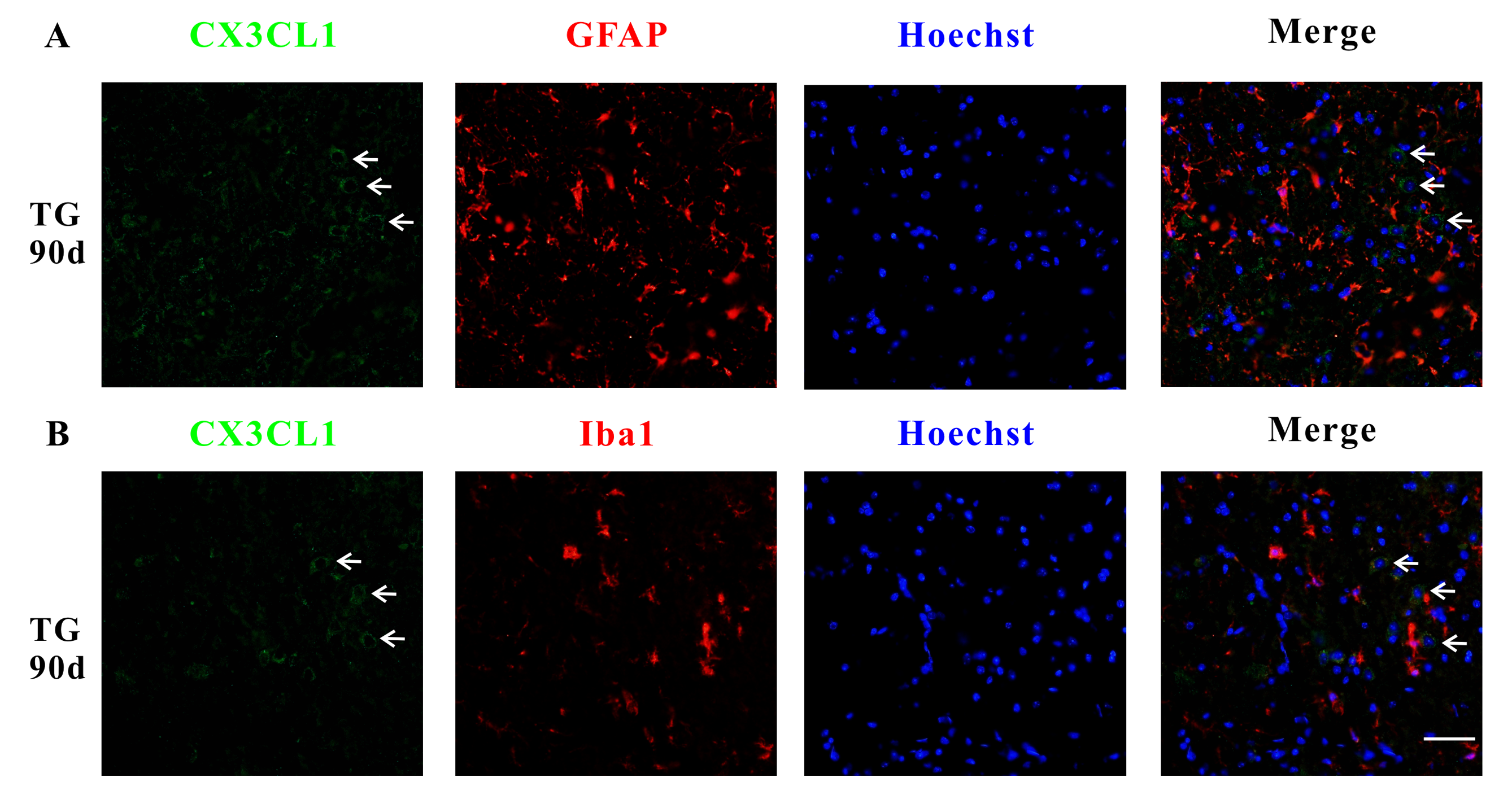

Supplement: Supplementary file 2 — Figure S2. The immunofluorescent staining of CX3CL1 on microglia or astrocytes. (A) Double immunofluorescent staining of CX3CL1 (green) and GFAP (red) positive astrocytes. (B) Double immunofluorescent staining of CX3CL1 (green) and Iba1 (red) positive microglia. Scale bar = 50 μm. (TIF 1941 kb) [file 40035_2018_138_MOESM2_ESM.tif]
